# Analysis of the Microbiota of Black Stain in the Primary Dentition

**DOI:** 10.1371/journal.pone.0137030

**Published:** 2015-09-04

**Authors:** Yue Li, Qian Zhang, Fangfei Zhang, Ruoxi Liu, He Liu, Feng Chen

**Affiliations:** 1 Department of Pediatric Dentistry, School of Stomatology, Peking University, Beijing, China; 2 Central Laboratory, School of Stomatology, Peking University, Beijing, China; University Hospital of the Albert-Ludwigs-University Freiburg, GERMANY

## Abstract

Black tooth stain is a characteristic extrinsic discoloration commonly seen on the cervical enamel following the contour of the gingiva. To investigate the relationship between black tooth stain and the oral microbiota, we used 16S rRNA gene sequencing to compare the microbial composition of dental plaque and saliva among caries-free children with and without black stain. Dental plaque and saliva, as well as black stain, were sampled from 10 children with and 15 children without black stain. Data were analyzed using the pipeline tool MOTHUR. Student’s *t*-test was used to compare alpha diversities and the Mann-Whitney U test to compare the relative abundances of the microbial taxa. A total of 10 phyla, 19 classes, 32 orders, 61 families and 102 genera were detected in these samples. Shannon and Simpson diversity were found to be significantly lower in saliva samples of children with black stain. Microbial diversity was reduced in the black stain compared to the plaque samples. *Actinomyces*, *Cardiobacterium*, *Haemophilus*, *Corynebacterium*, *Tannerella* and *Treponema* were more abundant and *Campylobacter* less abundant in plaque samples of children with black stain. Principal component analysis demonstrated clustering among the dental plaque samples from the control group, while the plaque samples from the black stain group were not and appeared to cluster into two subgroups. Alterations in oral microbiota may be associated with the formation of black stain.

## Introduction

Black tooth stain is a type of extrinsic discoloration of the tooth. It may be clinically diagnosed as pigmented, dark lines parallel to the gingival margin or as an incomplete coalescence of dark dots rarely extending beyond the cervical third of the crown[1]. Both primary and permanent teeth can be affected, with a reported prevalence of 1–20%[2].

Black stain (BS) is hard to be wiped off by daily cleaning via tooth brushing, and tends to re-form after professional scaling. BS can cause an aesthetic problem for individuals, but no associated impairment of dental health has been reported. Most epidemiological studies worldwide found that children with black-stained teeth had lower caries prevalence or experiences[3–7]. A study of a Brazilian population-based birth cohort also suggested that BS might be a protective factor for dental caries development[8].

Analysis of the black material in black-stained teeth indicates that it contains insoluble ferric salt, which is likely to be ferric sulfide, and a high content of calcium and phosphate[9, 10]. These studies suggested that the ferric sulfide might be formed by the reaction between hydrogen sulfide produced by bacterial action and iron in the saliva or gingival exudates. In one study, a group of 6-year-old Spanish children with black stain were found to have consumed a significantly higher proportion of iron supplements than children without black stain; an increased rate of iron supplementation was also found among the mothers of these children during pregnancy[11]. This study also reported higher consumption of specific foods rich in iron, such as vegetables, dairy products and eggs, in children with black stain. Furthermore, another study revealed a positive correlation between black stain and the concentration of iron in water sources[12]. These findings suggested that the formation of black tooth stain may be associated with ferric sulfide.

Chromogenic bacteria were proposed as an etiological factor in the production of black pigment. Periodontal pathogens such as *Porphyromonas gingivalis*, *Prevotella intermedia*, and *Prevotella nigrescens* are reported to be black-pigmented anaerobes in oral cavity[13]. Former studies assumed *Prevotella melaninogenica* was closely related to black tooth stain [14]. Traditional bacteriological examinations have implicated *Actinomycetes* as the predominant cultivable microorganisms found in black stain. However, almost 50% of oral bacteria are non-culturable. Saba et al. detected the presence of four distinct subspecies in dental plaque samples of 100 BS-affected children between 6 and 12 years old and 100 stain-free control subjects by PCR and electrophoresis. Significantly higher prevalences of *Actinomyces* and *Aggregatibacter actinomycetemcomitans* were observed in black stain compared to the plaque of the control group. *Porphyromonas gingivalis* and *Prevotella melaninogenica* were absent from the samples of both black stain and control subjects[15]. Furthermore, real-time PCR was performed to determine the microbial compositions of samples of black stain from 46 children aged from 3 to 10 years and non-discolored dental plaque from 47 counterparts without black stain. Plaque samples of the black stain group were found to contain higher numbers of *Actinomyces naeslundii* and lower numbers of *Fusobacterium nucleatum* and *Lactobacillus spp*. No significant differences were observed for *Aggregatibacter actinomycetemcomitans* and *Prevotella intermedia*[6]. The above studies indicate a unique microbiota in dental plaque with black stain. However, as the results were not unanimous and only a few selected species were evaluated, the relationship between microorganisms and black stain remains uncertain.

The traditional method of assessing oral microorganisms is bacterial culture. The disadvantage of this method is that almost 50% of oral bacteria are non-culturable. In addition, conventional culture methods are time-consuming. By contrast, molecular techniques have the advantage of detecting non-culturable bacteria as well as those that are difficult to cultivate. Besides, number of target bacteria for polymerase chain reaction (PCR) techniques is restricted, only expected species can be detected. In this study, we utilized next-generation sequencing of the bacterial 16S rRNA gene to detect oral microorganisms in children with or without BS. Compared to automated Sanger sequencing, which is considered as the ‘first-generation’ technology, NGS allows sequencing whole genomes without cloning in *E*. *coli* or any host cell, and can provides a larger number of reads and greater depth of coverage[16]. This is for the first time that NGS was applied to study black tooth stain. BS-relative bacteria were supposed to be found, which may offer more information for the research of the mechanism of black stain formation.

## Materials and Methods

### Subject Selection

**Fig 1:**
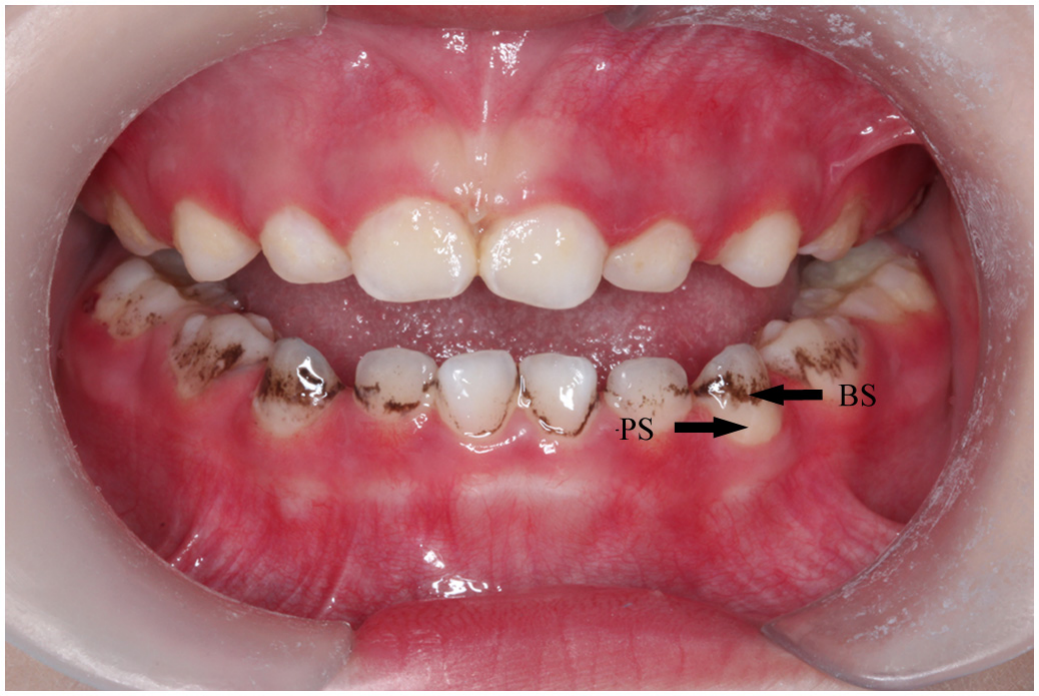
Primary dentition with more than six black-stained teeth. Black stain (BS) was found as dark pigmented lines or incomplete coalescence of dark dots on the cervical enamel following the contour of the gingiva. Samples of both black stain (BS) and dental plaque (PS) were collected from children with black stain.

A total of 25 caries-free children aged 4–5 years were recruited from the third kindergarten of the Chinese Academy of Sciences. 10 children with at least six black-stained teeth were included in group A, while children free of BS were in group B. No significant difference was found between groups involving age and gender. From each child in group A, samples of saliva (SS), plaque (PS) and black stain (BS) were obtained. Samples of supra-gingival plaque (PS) were collected from dental surface where there was no black material. Black stain (BS) was exactly the black material on tooth surface and was scraped off the teeth using dental curette. (Fig 1). From children in group B samples of saliva (SC) and plaque (PC) were obtained. Children in both groups were free of dental caries (dmfs = 0). None had systemic or infectious diseases, enamel hypoplasia or dentinogenesis imperfecta, or had used antibiotic drugs within the 2-week period before sample collection. The study design, protocol, and informed consent were approved by the Ethics Committee of Stomatological Hospital of Peking University (PKUSSIRB- 201311085). And written informed consent was obtained from the parents of all children enrolled in this study.

### Sample Collection

Samples were collected from both groups at approximately 8:40–11:20 in the morning at the third kindergarten. Informed consent was obtained from parents of participating children.

Subjects were asked to rinse their mouths with water before sample collection. Samples of dental plaque and black stain were collected using new metal curettes. An expectation of 1-2mg dental plaque and black stain were obtained. For children with black stain, we first collected their dental plaque on non-black stained surface, and then scraped their black stain into Eppendorf tubes. These children then had their teeth dry-brushed to collect stimulated whole saliva into a 50-ml centrifuge tube, for an expected amount of 1–2 ml. Saliva collection was performed within 5 min.

The plaque and black stain samples were immediately transferred to an Eppendorf tube containing 400 μl of sterile water. All samples were placed on ice before being transported to the laboratory.

### DNA Extraction

Saliva samples were centrifuged at 13,400g for 15 min and the precipitates were collected. Bacterial DNA was extracted from all samples using the QIAamp DNA Mini Kit (Qiagen, Hilden, Germany), following the manufacturer’s instructions, after initial treatment with lysozyme (20 mg/ml, 37°C for 1 h). The final quality of DNA was evaluated by Nanodrop2000 (Thermo, USA). A standard concentration of 10 ng/μl was prepared for each individual sample for all PCR assays.

PCR amplification of the V3-V4 region of the bacterial 16S rRNA gene was performed using universal primers (338F 5’-ACTCCTACGGGAGGCAGCA-3’, 806R 5’-GGACTACHVGGGTWTCTAAT-3’) incorporating a sample barcode sequence. The cycling parameters were as follows: initial denaturation at 94°C for 10 min; 30 cycles of denaturation at 94°C (30s), annealing at 65°C (30s), elongation at 72°C (30s); and final extension at 72°C for 10 min. PCR products were separated by 2% agarose gel electrophoresis and the fragments of ~500 bp were purified. Equal qualities of PCR products from each sample were pooled for further sequencing.

### Bioinformatic Analysis

Sixty samples were sequenced, and the raw data generated were analyzed using the pipeline tool MOTHUR[17, 18]. The multiplexed samples were deconvoluted based on the unique barcode assigned to each sample. The barcodes and primers were then trimmed off and sequences with a quality value of <20 and low-quality sequences were removed[19]. High-quality reads were clustered into operational taxonomic units (OTUs) at 97% similarity. Microbial diversity was estimated and the relative abundance of microbial taxa were calculated and compared. Student’s *t*-test was used to compare alpha diversities and the Mann-Whitney U test to compare the relative abundances of microbial taxa between two groups. Principal component analysis (PCA) was conducted according to the matrix of distance.

## Results

### Community distribution

The total number of samples was 60, from which 2 samples of PS were excluded due to the lack of sufficient sequences, resulting in 58 samples that were analyzed further. Totals of 10 phyla, 19 classes, 32 orders, 61 families and 102 genera were detected in these samples.

**Fig 2:**
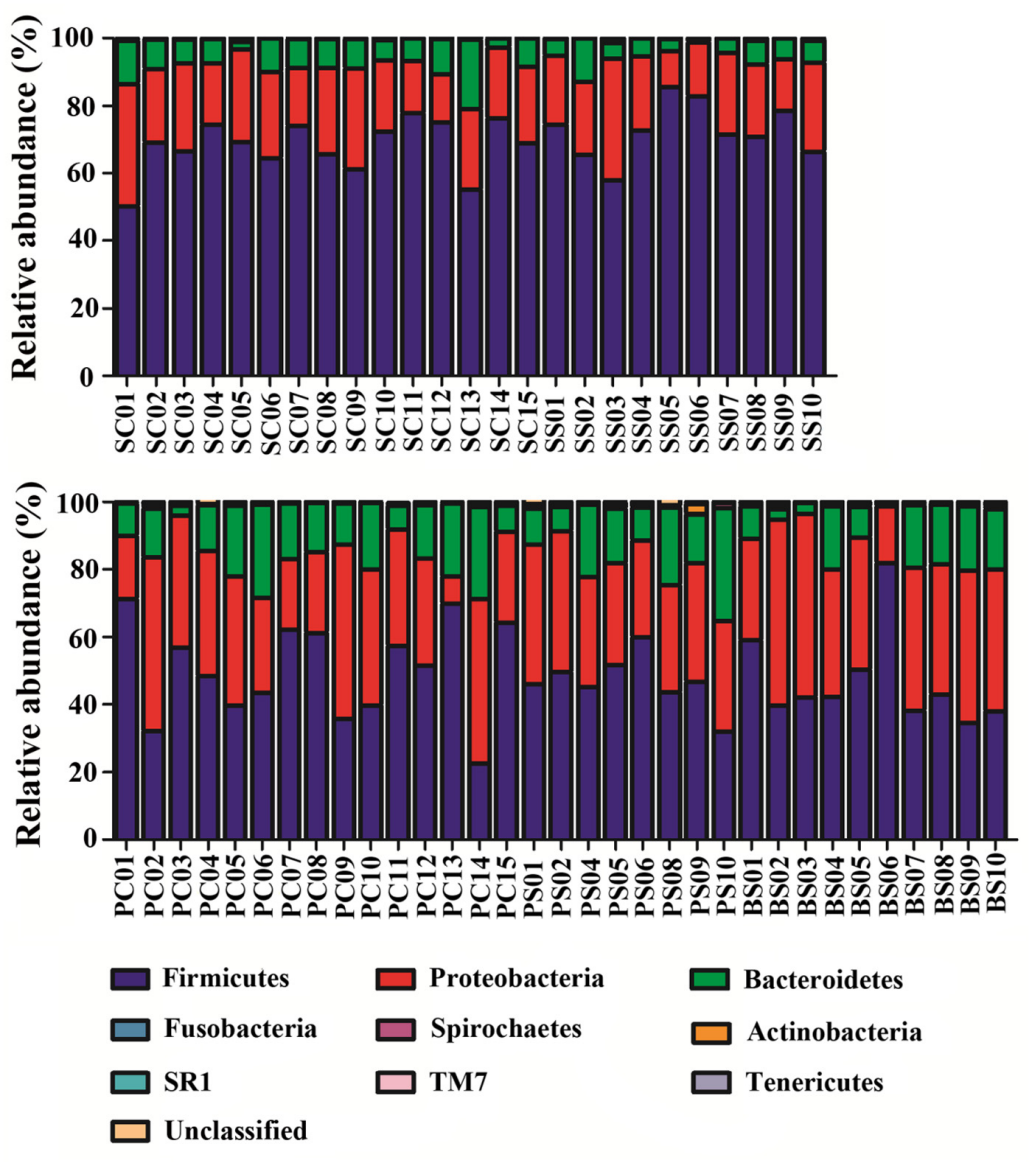
Relative abundance of bacterial composition at phylum level in saliva and plaque as well as black stain (BS) samples.

In the PC, PS and BS samples, the 3 most abundant of the 10 phyla were *Firmicutes*, *Proteobacteria* and *Bacteroidetes*, which represented more than 95% of the total sequences. In samples of saliva, SC and SS, the aforementioned three phyla also constituted the majority of the sequences. *Firmicutes* represented more than 50% of the total sequences (Fig 2).

At the genus level, among the 102 genera detected, the five most abundant in plaque and BS samples were *Streptococcus*, *Neisseria*, *Burkholderiales unclassified*, *Capnocytophaga* and *Neisseriaceae unclassified*. These constituted more than 60% of the total sequences. In saliva, *Streptococcus*, *Neisseria* and *Capnocytophaga* represented more than 60% of the total sequences (S1 Fig).

### OTU diversity

**Fig 3:**
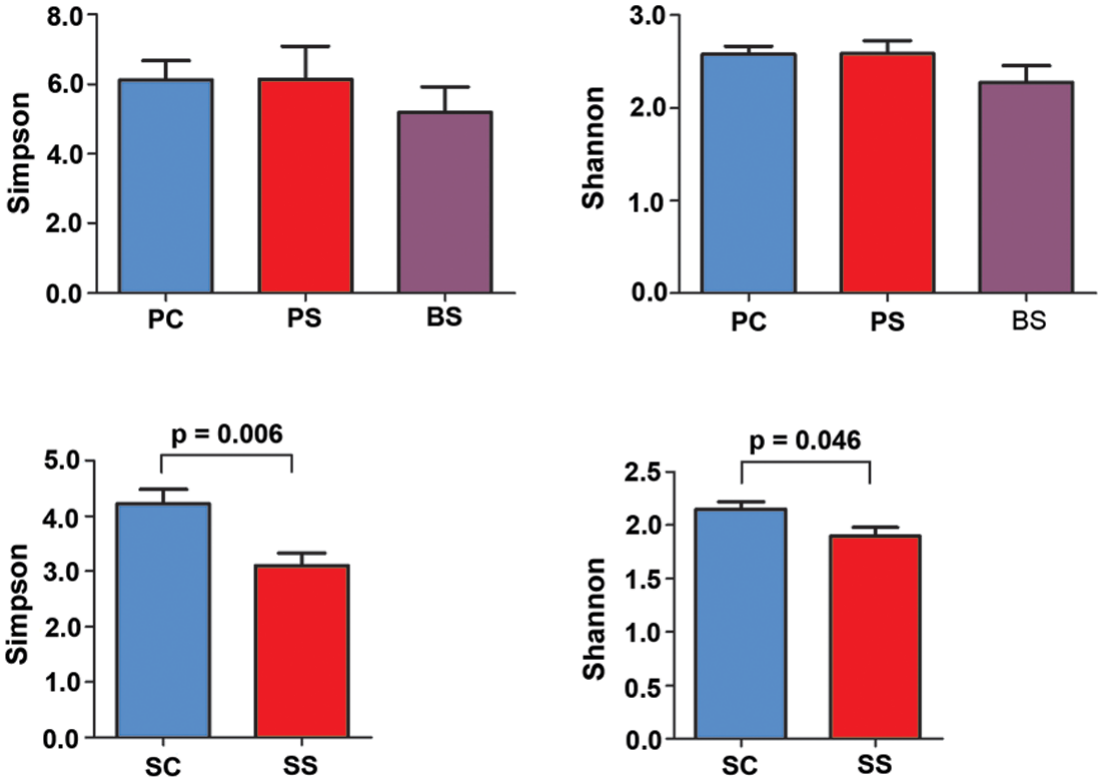
Microbiota diversity as calculated by Shannon and Simpson index. The Shannon and Simpson index values were significantly higher in salivary samples of the control group (SC) than the black stain group (SS). A tendency towards a reduction in the Shannon and Simpson index was observed in samples of black stain (BS) compared to dental plaque from the control (PC) and BS groups (PS); however, the difference was not significant. The error bars indicate mean with standard error.

In total, 3475 OTUs were obtained at a 97% similarity level. The richness of the total amount of bacteria was estimated by ACE and Chao. The diversity of the microbiota was estimated by Shannon and Simpson indices[20]. Student’s *t*-test was used to compare alpha diversities between groups. The Shannon and Simpson index showed a significant difference between SC and SS samples. The saliva microbial diversity in children with BS was lower than that in children without BS. Though there was no significant difference among the PC, PS and BS groups, the Shannon and Simpson index suggested a tendency towards a reduction in the BS group (Fig 3).

**Fig 4:**
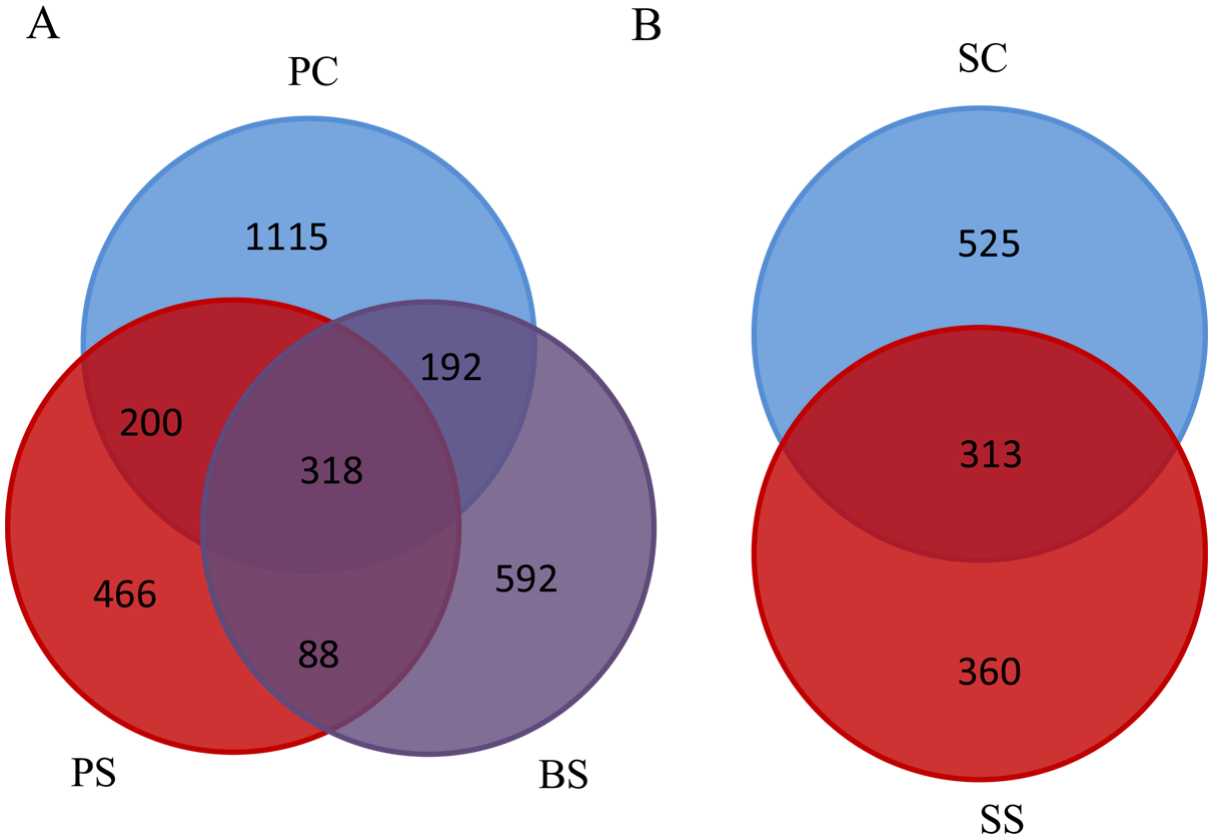
Venn diagram of the number of OTUs common/unique to the black stain and control groups. The overlapping areas represent the number of OTUs shared by the counterpart samples. A: A total of 2971 OTUs were detected in black stain (BS) and the dental plaque of the black stain group (PS) and control group (PC). Only 318 OTUs were shared by PS, PC and BS, while 1115, 466, and 592 OTUs were unique to the respective groups. B: A total of 1198 OTUs were detected in the salivary samples of the black stain group (SS) and control group (SC). Only 313 OTUs were shared by the SC and SS groups, and 525 and 360 OTUs were unique to the respective groups.

After removing the duplicate sequences, 2971 OTUs were measured in the PC, PS and BS groups, with 318, 200, 88,192 OTUs shared by samples PC/PS/BS, PC/PS, PS/BS, PC/BS, respectively. Unique measurements of OTUs in the PC, PS, and BS groups were 1115, 466 and 592, respectively. The number of OTUs in saliva samples was 1198, with 313 shared by SC and SS, and 525 and 360 OTUs unique to the respective groups (Fig 4).

### Taxonomic analysis

**Fig 5:**
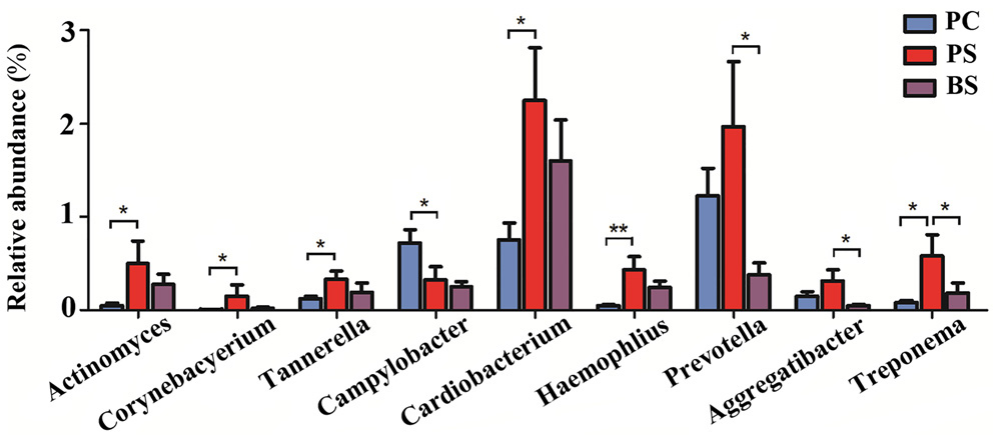
Comparisons of microbiota that presented significantly different contents in dental plaque of the black stain group (PS) and control group (PC) and black stain (BS) at genus level. *P<0.05; **P<0.01.

After removing the 2 samples of poor quality, we compared microbiota between groups at various levels using the Mann-Whitney U test. Each of the 10 phyla, 19 classes, 32 orders, 61 families and 102 genera was compared between PC and PS, PS and BS, SC and SS. There were significant differences between the PC and PS groups at the following levels: *Actinobacteria* (phylum), *Actinobacteria*, *Gammaproteobacteria*, and *Epsilonproteobacteria* (class), *Actinomycetales*, *Cardiobacteriales*, *Pasteurellales*, and *Campylobacterales* (order), *Actinomycetaceae*, *Cardiobacteriaceae*, *Pasteurellaceae*, *Campylobacteraceae*, *Corynebacteriaceae*, and *Porphyromonadaceae* (family) (S3 Fig), *Actinomyces*, *Cardiobacterium*, *Haemophilus*, *Campylobacter*, *Corynebacterium*, and *Tannerella* (genus) (Fig 5).

There were significant differences between the PS and BS groups at the following levels: *Spirochaetes* (phylum), *Spirochaetes* (class), *Spirochaetales* and *Pasteurellales* (order), *Spirochaetaceae*, *Pasteurellaceae* and *Prevotellaceae* (family) (S3 Fig), and *Treponema*, *Aggregatibacter* and *Prevotella* (genus) (Fig 5). Bacterial genera that were more abundant in children with BS are listed in Table 1.

**Table 1:** Bacterial phylotype that were more abundant in children with black stain.

Bacterial phylotype	Characteristics	References
Actinomyces	Produce H_2_S	[21]
	Pigmentation	[22]
	Normal oral flora	
Tannerella	Periodontitis(*Tannerella forsythia*)	[27]
Treponema	Periodontal diseases	[26]
Corynebacterium	Dental calculi (C. *matruchotii*)	[25]
	Opportunistic pathogen	
Cardiobacterium	Endocarditis	[23]
	Normal flora	
Haemophilus	Respiratory tract infection	[24]
	Normal flora	

We also compared the salivary microbiota composition of the two groups and found differences in the following microorganisms: *Bacteroidetes* (phylum), *Flavobacteria* (class), *Flavobacteriales* (order), *Flavobacteriaceae* (family) and *Capnocytophaga* (genus) (S4 Fig).

### Principal Component Analysis (PCA)

**Fig 6:**
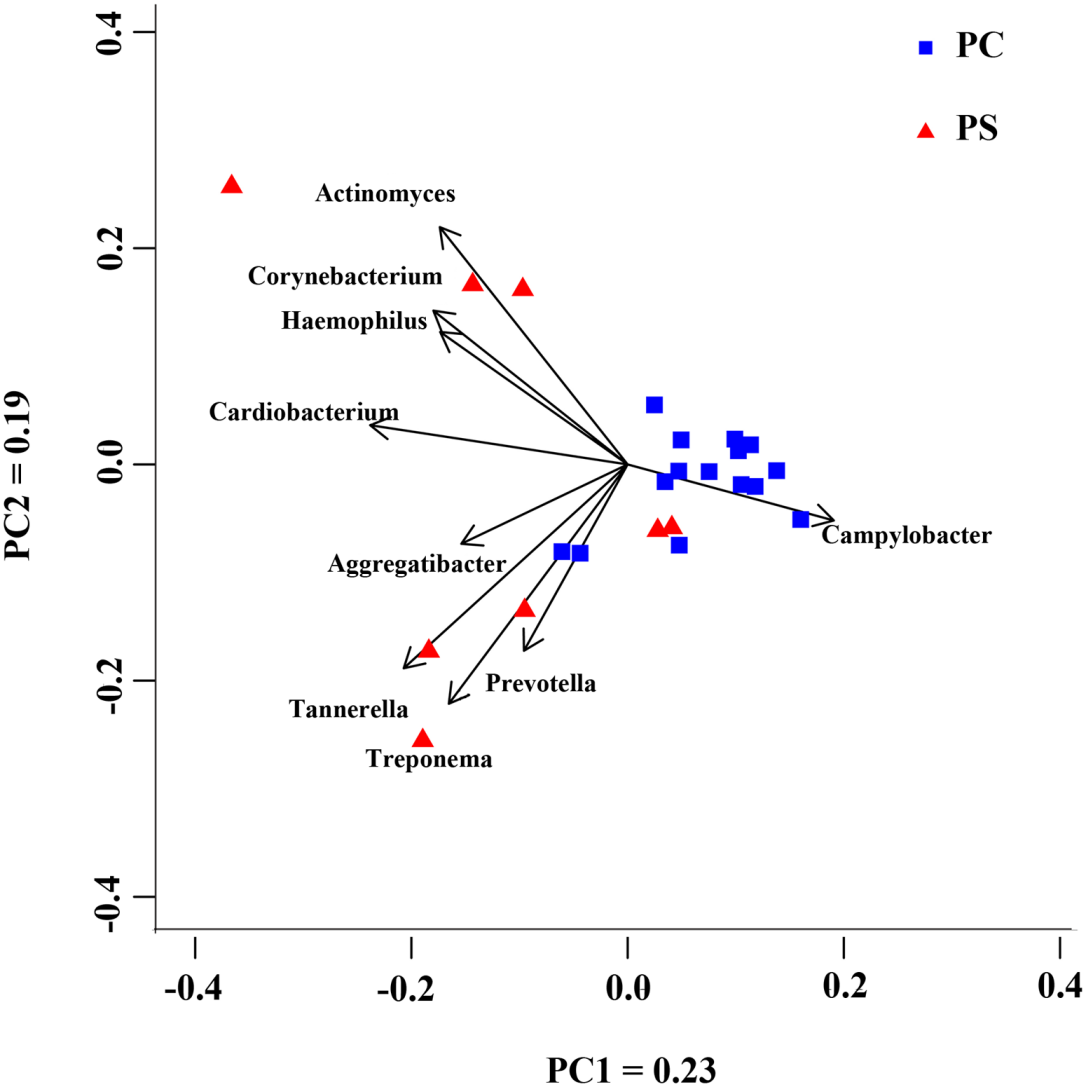
Principal component analysis of the bacterial genera detected in the dental plaque of the control group (PC) and black stain group (PS). The PC samples clustered together, with two samples mixed into the PS cluster, while the PS samples were more dispersive and seemed to cluster into two subgroups.

To classify the bacteria into two groups, a PCA was implemented (Fig 6) based on genus information. The PC samples appeared to cluster together, with two samples mixed in the PS cluster, while the PS samples were more dispersive and seemed to cluster into two subgroups. This implied there may be complex mechanisms for black stain formation, but a study involving a larger sample size would be required to confirm this.

## Discussion

This study used next-generation sequencing of the bacterial 16S rRNA gene to evaluate the oral microbiota in children with and without black stain. The salivary microbiota diversity in children with black stain was significantly lower than in children without black stain. Regarding the plaque microbiota, although the Shannon and Simpson index showed no significant differences among PC, PS and BS groups, the microbial diversity tended to be lower in BS samples compared with PC and PB samples. This suggested that microbial diversity is reduced in children with black stain, which might be associated with black stain formation.

Former studies had found the relative abundance of *Actinomyces* was higher in children with black stain compared to children without black stain[6, 15]. Our study confirmed these findings. Hence it is possible that *Actinomycetes* is involved in the deposition of BS. *Actinomyces* belong to the resident oral microbiota of supra-gingival plaque. Some *Actinomyces* strains produce hydrogen sulfide[10, 21], which can result in ferric sulfide formation in the presence of iron in saliva or gingival exudates. Sarkonen et al. found that *A*. *odontolyticus*, *A*. *graevenitzii* and *A*. *radicidentis* produce pigments, with colors ranging from brown to black, when cultured on rabbit slaked blood agar[22]. However, this pigmentation may not be true pigment production, but rather a result of medium decomposition. Nevertheless, the proportions of *Actinomyces* could account for different susceptibilities to extrinsic black stain formation.

*Cardiobacterium*, *Haemophilus*, *Corynebacterium*, *Tannerella* and *Treponema* were also more abundant in plaque samples from children with black stain. These bacteria may not produce pigments or hydrogen sulfide. However, the overall plaque genome suggests that *Actinomyces* may not be the only microorganism involved in black stain formation. Alterations in the abundance of several other species could provide an environment that facilitates black stain deposition on the enamel surface. *Corynebacterium*, *Cardiobacterium*[23] and *Haemophilus*[24] were not previously reported to be associated with extrinsic black stain. *Corynebacterium matruchotii* was supposed to be the main *Corynebacterium* species in the oral cavity of humans, and is associated with the formation of dental calculi[25]. On the other hand, *Campylobacter* was detected in this study to be more abundant in PC than in PS. These genus are commonly found in oral cavity. As a more sensitive and culture-independent method, 16S rRNA gene sequencing makes it possible to detect previously undetected species, but whether these microorganisms are related to black stain formation remains to be determined.

Saba et al. detected the periodontal pathogen *A*. *actinomycetemcomitans* at a higher frequency in the black stain group and discussed the potential future risk of developing periodontitis[15]. Using real-time PCR, Heinrich-Weltzien et al. found no significant difference in the prevalence of *A*. *actinomycetemcomitans* and *P*. *intermedia* between black stain group and control group[6], which is consistent with our results. In addition, we detected *Tannerella* and *Treponema* at a higher prevalence in the BS group (Table 1). Both *Tannerella* and *Treponema* are related to periodontal disease[26, 27]. Sakai et al. evaluated the prevalence of putative periodontal pathogens (*Aggregatibacter actinomycetemcomitans*, *Porphyromonas gingivalis*, *Prevotella nigrescens*, *Treponema denticola*) in the saliva of children with mixed dentition, and found a high percentage of children harbored at least one of these four pathogens without signs of periodontal disease[28]. Further studies should analyze the role of periodontopathogenic bacteria in BS and the susceptibility of children with black stain to the development of periodontal disease.

The presence of black stain has been reported to be associated with low frequency of caries, for as-yet-unknown reasons. Slots proposed that the low caries occurrence might be due to lower numbers of *Streptococcus*, one of the main pathogens of dental caries[14]. According to Heinrich-Weltzien, counts of *S*. *mutans* and *S*. *sobrinus* were not significantly different between samples of BS and non-discolored plaque in both caries-free and -affected children, but counts of *Lactobacillus spp*. were higher in non-discolored plaque samples[6]. In our study, no significant difference of *Streptococcus* among groups was found. Reid and Beeley suggested that the reduction in the incidence of caries in individuals with black extrinsic tooth stains is due to the calcium and phosphate content of the biofilm of the stain[10]. Considering the multifactorial nature of caries disease, the hypothesis that the microbial composition of BS might be associated with the lower caries experience of children is questionable.

In conclusion, black stain in the primary dentition might be more correlated with differences in the microbiota in dental plaque than in saliva. Children with black stain exhibit a reduced microbial diversity and black stain formation may be related to alteration of the plaque microbiota. *Actinomyces* was more abundant in plaque samples of children with black stain and thus might be a causative agent. Alterations in the abundance of other species might provide an environment that enables black stain formation and deposition onto the surface of enamel. Furthermore, the role of these microorganisms in black stain formation and the association of black stain with periodontitis and caries require further research.

This is the first time that next generation sequencing has been applied to assess the microbiology of black tooth stain. It provided us a broader view of the microbiota in black stain from the aspect of bacterial composition compared to former studies using PCR or rt-PCR. However, the descriptive results cannot confirm the mechanisms of black stain formation. We need further studies to analyze functions of these bacteria. On the other hand, our sample size is relatively small, we will collect more samples in our clinic and kindergarten for further study to verify our conclusion.

The English in this document has been checked by at least two professional editors, both native speakers of English. For a certificate, please see: http://www.textcheck.com/certificate/ZAboUD

## Supporting Information

S1 Fig Relative abundance of bacterial composition at genus level in saliva and plaque as well as black stain (BS) samples. (TIF)

S2 Fig Microbiota diversity was calculated by Chao 1 and Ace index. No significant difference was found (P>0.05).
(TIF)

S3 Fig Comparisons of microbiota that presented significantly different contents in dental plaque of the black stain group (PS) and control group (PC) and black stain (BS) at phylum, class, order and family level. *P<0.05; **P<0.01.
(TIF)

S4 Fig Microbiota that presented significant difference in saliva samples between black stain group and control group at different level. (TIF)

S5 Fig Rarefaction curves of each sample. An average of 4638 reads was randomly drawing out from each sample for subsequent OTU analysis.
(TIF)

S1 Table Baseline data of the two groups of children. (DOCX)
