# Supplementary figures and images for: Analysis of the Microbiota of Black Stain in the Primary Dentition
Source: PLoS One. 2015 Sep 4;10(9):e0137030. doi: 10.1371/journal.pone.0137030 (PMC4560370; doi:10.1371/journal.pone.0137030)

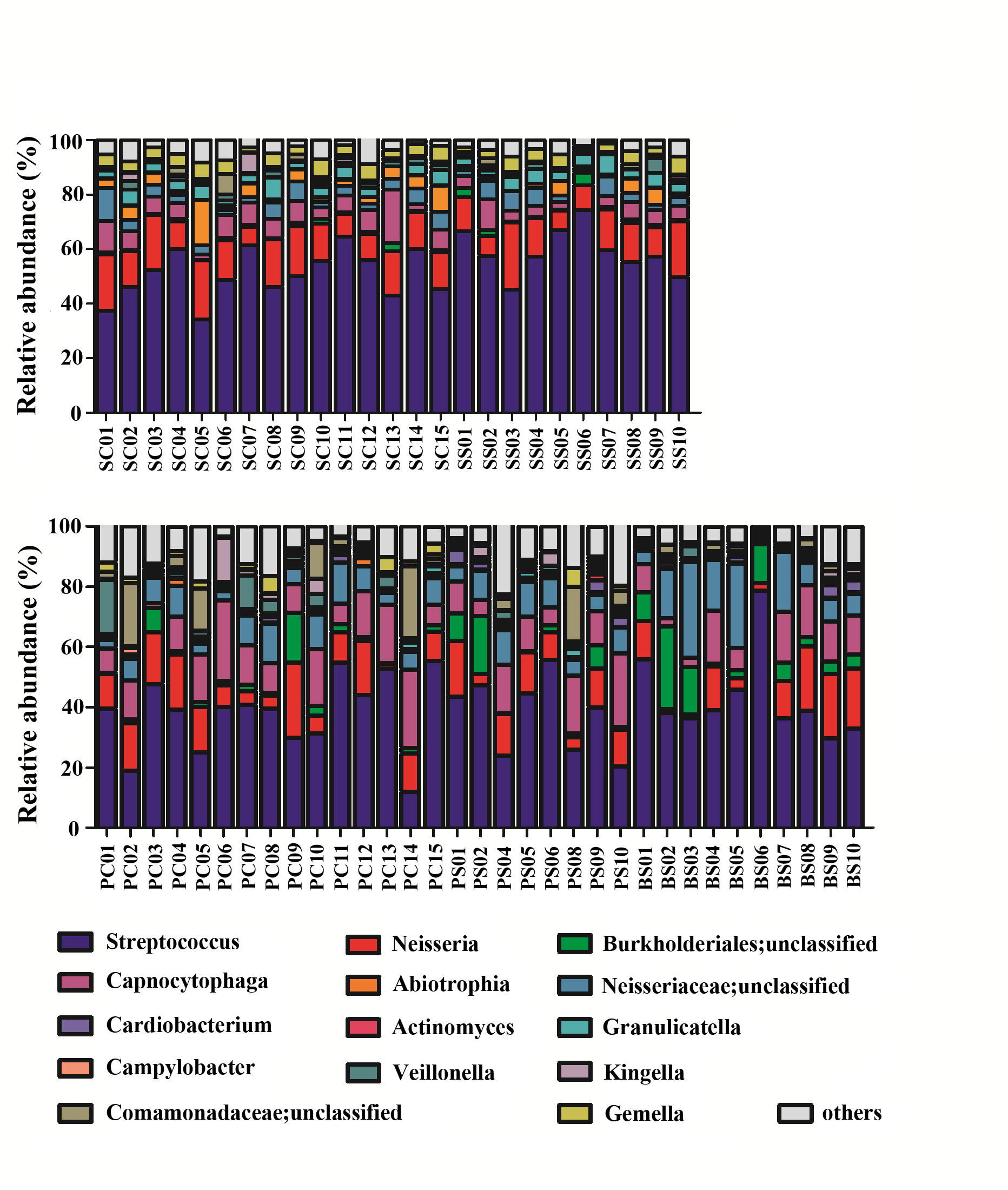

Supplement: S1 Fig — (TIF) [file pone.0137030.s001.tif]

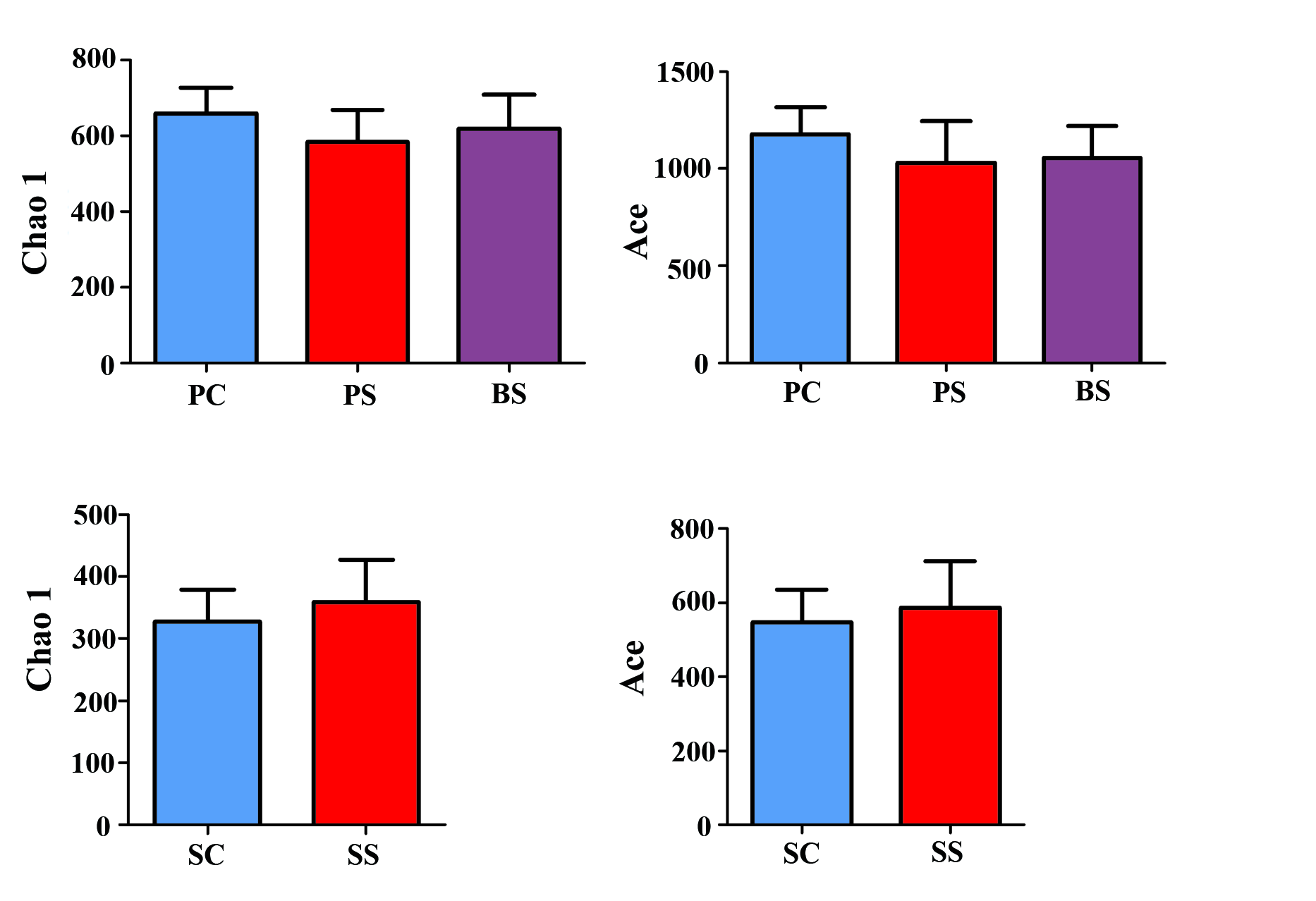

Supplement: S2 Fig — No significant difference was found (P>0.05). (TIF) [file pone.0137030.s002.tif]

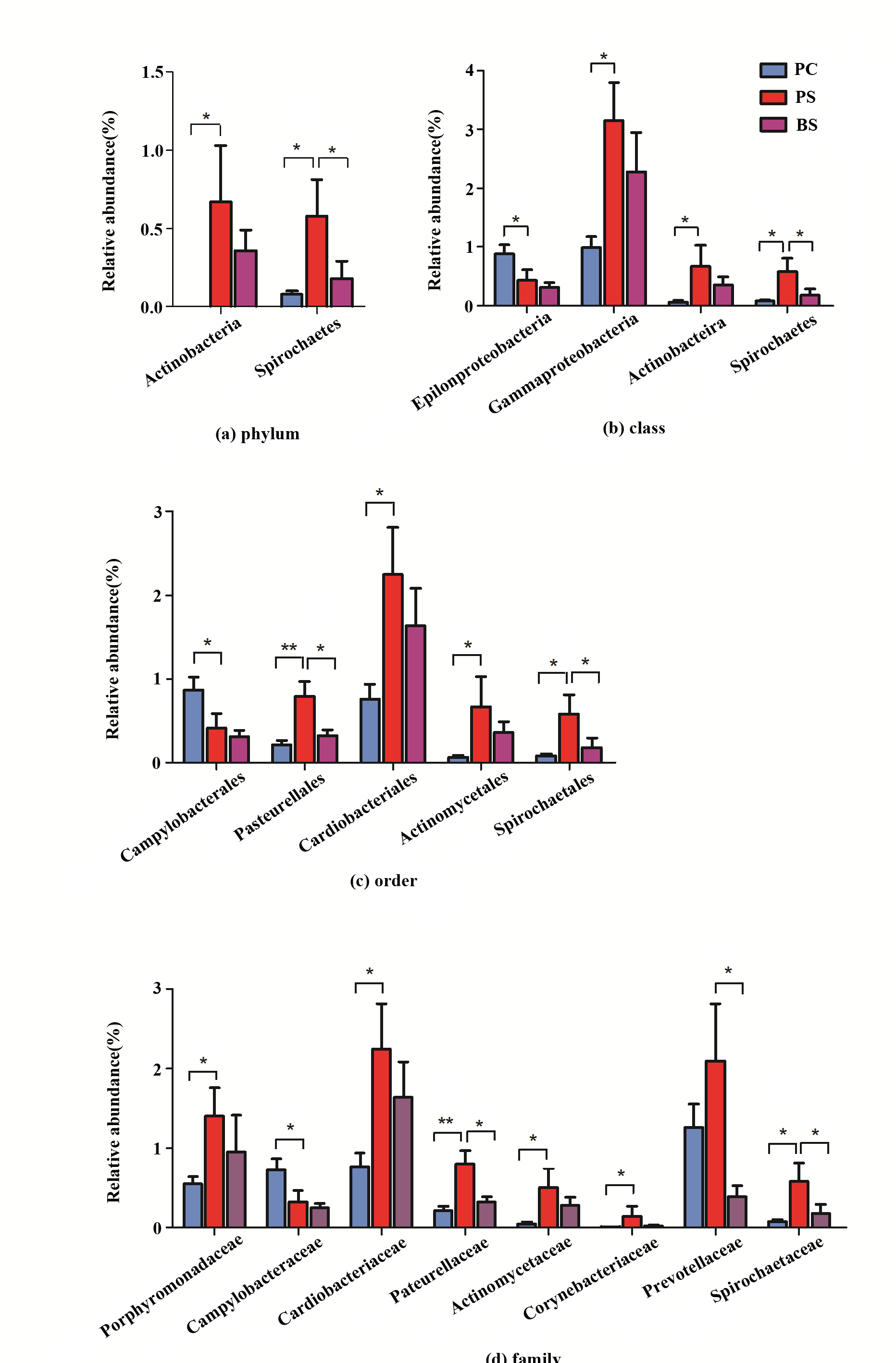

Supplement: S3 Fig — *P<0.05; **P<0.01. (TIF) [file pone.0137030.s003.tif]

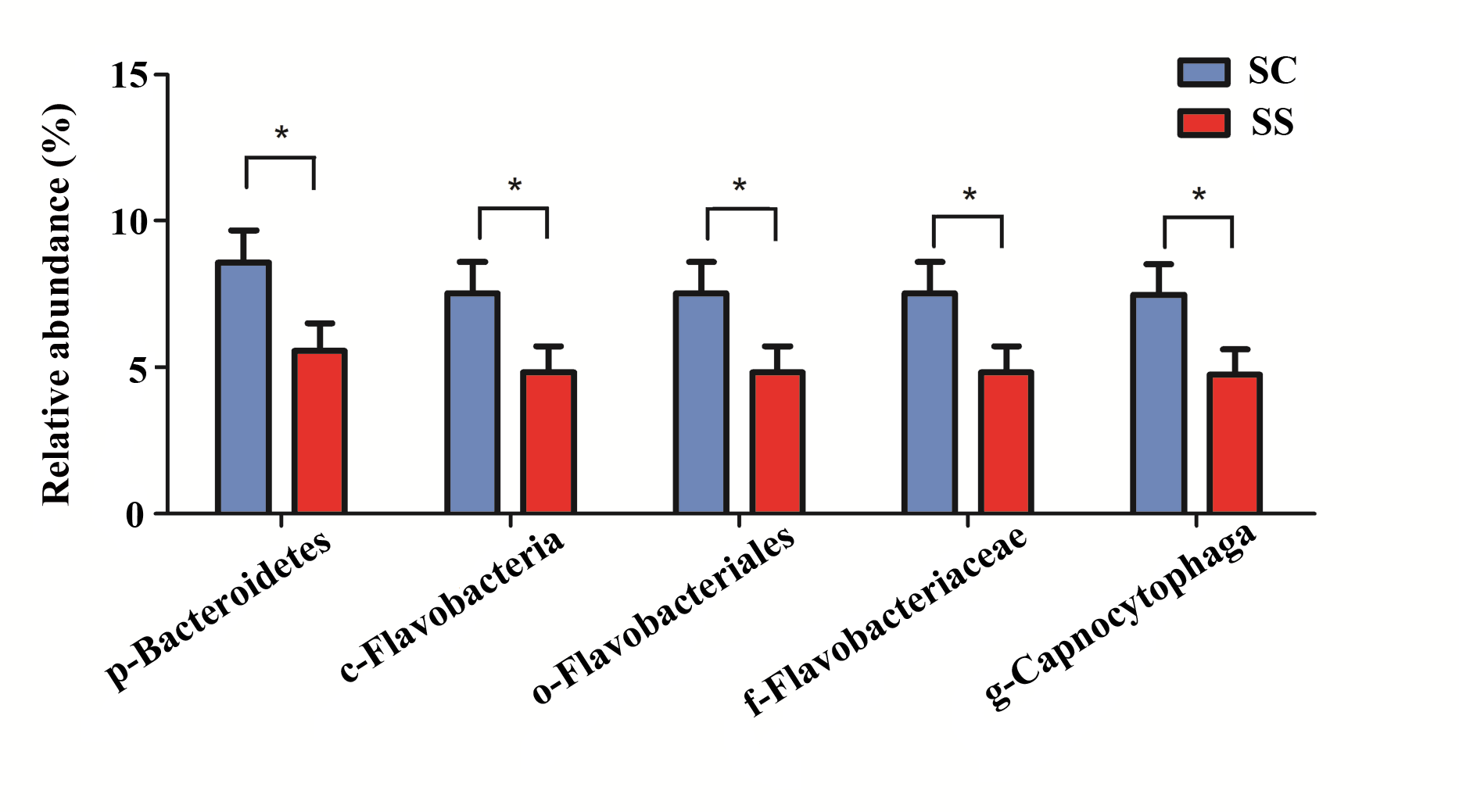

Supplement: S4 Fig — (TIF) [file pone.0137030.s004.tif]

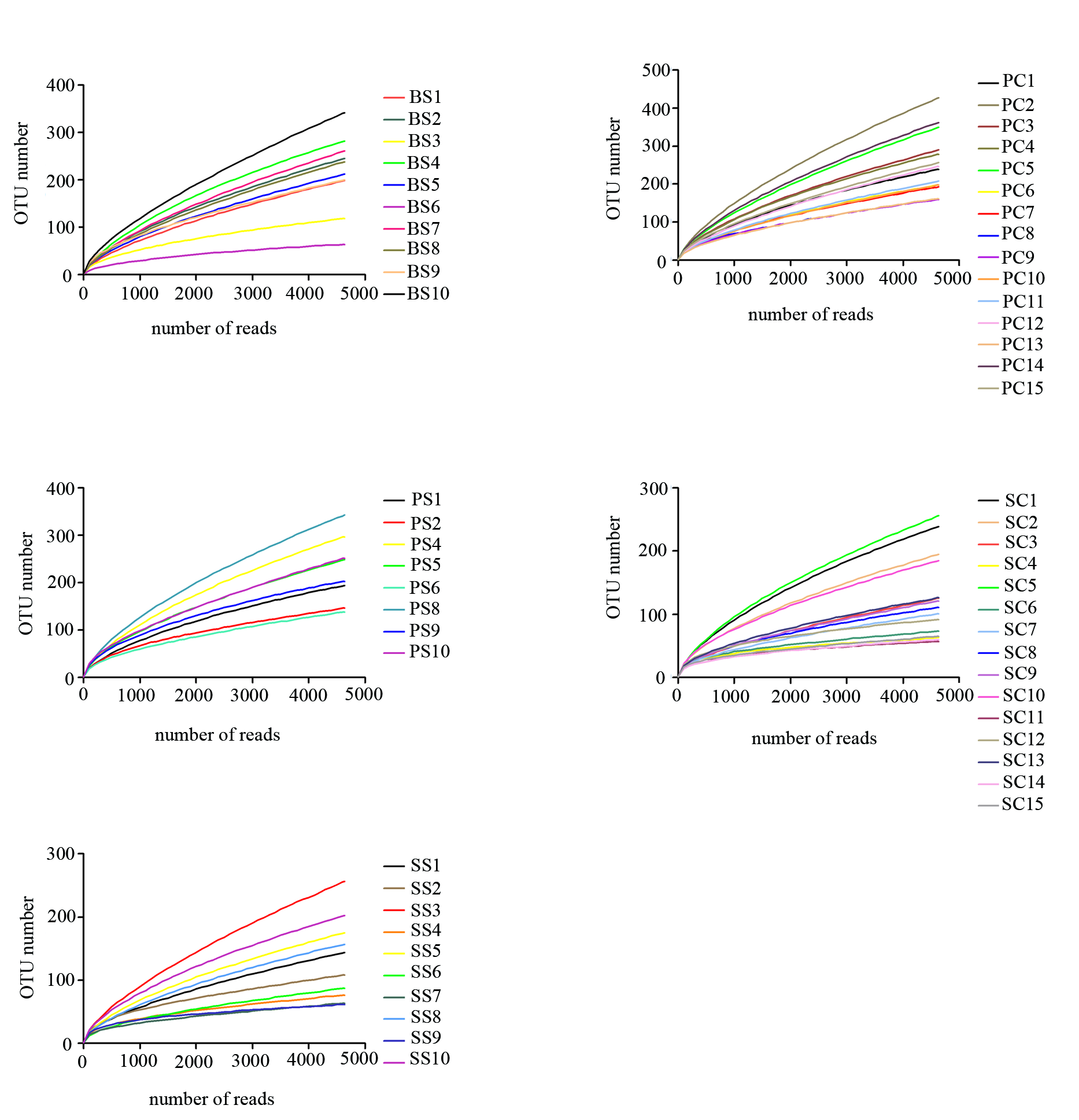

Supplement: S5 Fig — An average of 4638 reads was randomly drawing out from each sample for subsequent OTU analysis. (TIF) [file pone.0137030.s005.tif]
