# Supplementary material for: Analysis of the Microbiota of Black Stain in the Primary Dentition
Source: PLoS One. 2015 Sep 4;10(9):e0137030. doi: 10.1371/journal.pone.0137030 (PMC4560370; doi:10.1371/journal.pone.0137030)
Supplement: S1 Table — (DOCX) [file pone.0137030.s006.docx]

Table S1. Baseline data of the two groups of children

| Group | Number | Age(medium) | Male/Female | Caries |
| --- | --- | --- | --- | --- |
| Black stain | 10 | 4-5y(4.08) | 4/6 | None |
| Control | 15 | 4-5y(4.09) | 9/6 | None |
